# Early diagnostic markers in predicting the severity of dengue disease

**DOI:** 10.1007/s13205-022-03334-9

**Published:** 2022-09-09

**Authors:** Errol Moras, Basavaprabhu Achappa, B. V. Murlimanju, G. M. Naveen Raj, Ramesh Holla, Deepak Madi, Nikhil Victor D’Souza, Soundarya Mahalingam

**Affiliations:** 1 Intern, Kasturba Medical College, Mangalore, Manipal Academy of Higher Education, Manipal, Karnataka India; 2 Department of Internal Medicine, Kasturba Medical College, Mangalore, Manipal Academy of Higher Education, Manipal, Karnataka India; 3 Department of Anatomy, Kasturba Medical College, Mangalore, Manipal Academy of Higher Education, Manipal, Karnataka India; 4 Kasturba Medical College, Mangalore, Manipal Academy of Higher Education, Manipal, Karnataka India; 5 Department of Community Medicine, Kasturba Medical College, Mangalore, Manipal Academy of Higher Education, Manipal, Karnataka India; 6 Department of Paediatrics, Kasturba Medical College, Mangalore, Manipal Academy of Higher Education, Manipal, Karnataka India

**Keywords:** Dengue fever, Ferritin, Gall bladder diseases, Severe dengue

## Abstract

The aim of the present study was to determine whether the serum ferritin, the biomarker of an acute phase reactant and the gall bladder wall edema, an early indicator of capillary leakage can predict the severity of dengue fever. This study included 131 patients, who were between the age group of 18–80 years. The patients presented to our department with an acute illness, within the first four days of high temperature. The statistical analysis of this study was performed by using the Chi-square and independent Student’s *t* tests. The diagnostic markers are considered statistically significant, if the serum ferritin level is higher than 500 ng/ml and the gall bladder wall thickness is more than 3 mm. The present study observed that, 39 patients (89%) who had severe dengue (*n* = 44) revealed a significant gall bladder wall thickening, and this correlation was significant statistically (*p* < 0.000). It was also observed that, the ferritin levels have a highly significant positive correlation with the severity of dengue. The severe dengue patients had a mean ferritin level of 9125.34 μg/l, whereas the non-severe group had 4271 μg/l. This comparison was also statistically significant, as the *p* value was 0.003. We report that the serum ferritin levels have a highly significant positive correlation with the severity of dengue. The gall bladder wall edema during the third and fourth day of the illness was also associated with severe dengue. However, diffuse gall bladder wall thickening and high serum ferritin levels are also reported in various other conditions and their exact cause have to be determined by the correlation of associated clinical findings and imaging features.

## Introduction

Dengue is a febrile viral infection, which ranges between the self-limiting, milder form to the life-threatening, severe condition. There are several mechanisms, which explain the etiopathogenesis of severe dengue, which include antibody-dependent enhancement (ADE) (Avirutnan et al. 2006, 2010), overwhelming of stimulation of memory T cells (Avirutnan et al. 2007), and exaggeration of the proinflammatory cytokine response (Vaughn et al. 2000; Libraty et al. 2002). The fatty acid synthase 1 (FAS1) of adult *Aedes aegypti* mosquito has the highest similarity to the human FAS, concerning the amino acid and enzymatic domains. The *A. aegypti* fatty acid synthase 1 (AaFAS1) may play a role in the transmission of dengue (Chotiwan et al. 2022). During the high temperatures of climate, the immune dynamics of dengue are highly predominant and may become asynchronous (García-Carreras et al. 2022). There are different mechanisms for the dengue hemorrhagic fever (DHF) and shock syndrome of dengue (DSS), which are often fatal (Medin et al. 2005; Carr et al. 2003; Bosch et al. 2002). During the initial days of fever, there will be a window period in which the IgM antibodies may be negative. However, the NS1 antigen can still detect the infection of dengue. At this period, the biochemical and hematological parameters, which point towards the other differential diagnoses, are also absent. If there is a dilemma about the management, one can use further molecular methods like RT-PCR and probe based real-time RT-PCR for the early diagnosis of dengue. The singleplex real-time RT-PCR assay was recently invented for the pan-dengue virus detection and quantification (Songjaeng et al. 2022).

Since there is no available treatment for the dengue at present, it is necessary to develop some prognostic markers, which can predict the severity of dengue (Deubel et al. 1990). There is marked elevation of serum ferritin levels in dengue than in other viral or bacterial infections. The higher serum ferritin level is associated with the severe form of the disease in the pediatric age group. If the serum ferritin levels are more than 500 μg/l, it is defined as the hyperferritinemia (Senjo et al. 2018). The hyperferritinemia is observed in diseases, which have hyperactivation of the immune system and activation of macrophages. The elevated serum ferritin level suggests a greater risk of developing the complications, as this is associated with the hepatic injury (Zhang et al. 2021). The serum ferritin level measured either on the fourth or fifth day of fever, predicts the chances of dengue infection (van de Weg et al. 2014). The patients of dengue, associated with the elevated serum ferritin levels have to be monitored for the severe form of the disease, which leads to the activation of immune system and abnormal coagulation (Petchiappan et al. 2019). Ferritin is an acute-phase reactant, which is produced by the macrophages, hepatic cells, and monocytes (Wang et al. 2010). The high ferritin levels are associated with the pro-inflammatory cytokine release (Ruddell et al. 2009). The ferritin binds to the kininogen and blocks the bradykinin release. It is produced as a defense mechanism to protect the host. The higher level of serum ferritin is associated with the low platelet count and rising levels of liver enzymes, along with the activation of coagulation and fibrinolytic systems. It was reported that, some patients with severe dengue develop symptoms, which are similar to the hemophagocytic lymphohistiocytosis (HLH), suggesting a similar pathophysiology (van de Weg et al. 2014).

The dengue dyspepsia syndrome is the next most common complaint, besides the fever. It is due to the gastric mucosal and gall bladder wall congestion because of the capillary leakage of plasma (Adil et al. 2020). The gall bladder wall thickening is one of the commonly detected ultrasonographical observation in dengue fever. It has been described that, ultrasonography is instrumental in screening the patients progressing to the critical phase of dengue. The ultrasound can measure the gall bladder wall thickness. However, there is paucity of literature in determining, whether the gallbladder wall thickness in dengue fever is an early predictor of the severe form of dengue (Parmar et al. 2017). The ultrasound, as a prognostic indicator could be used to determine the patients who are at risk for entering the critical phase of dengue. Early identification of such patients could improve the overall case outcomes and enable the more productive use of hospital resources. However, there are very few studies performed regarding the relation of gall bladder wall thickening with the clinical manifestation of severe dengue, in spite of severe dengue predominating in the hospitalized patients (Fonseca-Portilla et al. 2021). In this context, the objective of this present study was to determine whether the biomarker, serum ferritin and edema of the gall bladder can predict the severity of dengue fever.

## Materials and methods

This was a descriptive study performed in the department of internal medicine of two different tertiary referral hospitals. This research was scrutinized and approved by the ethics committee of our institution. The written and informed consent was taken from all the patients of this study in their preferred language. The calculation of sample size was done by the sensitivity of ferritin for predicting the severe dengue, which was 77%, with a precision of 5% (*d* = 0.05). This was found to be 131 with a confidence interval of 95% as per the Buderer’s formula. Among the 1200 patients between 18 and 80 years of age, 131 patients who presented to us within the 4 days of acute febrile illness only were included in this study. They were qualitatively screened for the detection of dengue NS1 antigen by rapid immuno-chromatographic test (NS1 antigen card by J. Mitra & Co Pvt. Ltd). These were then confirmed with the help of the serological test, dengue IgM antibody titer by ELISA (PanBio dengue IgM capture ELISA kit). Those who were found positive were included in this study. We do this as a part of our protocol at our institution, as the national guidelines do not accept the dengue NS1 antigen card test, as the gold standard for the diagnosis of dengue. This needs to be confirmed by the IgM ELISA.

All the admitted patients had their vitals monitored and were looked for complications like hypotension, bleeding, narrow pulse pressure, evidence of capillary leakage in the form of effusion, ascites and edema. The relevant investigations were done serially on case to case basis. The ‘H’ score was calculated, which is a predictor of hemophagocytic lymphohistiocytosis. Either on the third or fourth day of fever, the serum ferritin level (electrochemiluminescence immunoassay analyser, ECLIA, Cobas 6000, Roche company) and the edema of gall bladder were checked. The same radiologist performed all the ultrasound abdomen examinations, to avoid the inter observer bias. The serum ferritin level is considered as high if it is more than 500 ng/ml (Senjo et al. 2018) and more than 3 mm of gall bladder wall thickness was considered as the significant edema (Adil et al. 2020).This study did not include the controls as the serum ferritin levels, and ultrasound abdomen were not done in other cases of fever, which were negative for the NS1 antigen card test. These were not performed as they would incur additional costs to the patients, and this was not a funded research. This can be considered as one of the limitations of this study, as the correlation between the controls and cases with respect to the serum ferritin levels, and ultrasound abdomen was not performed. The statistical analysis of the data was performed by using the independent Student’s *t* test and Chi-square test. The comparison was considered statistically significant if the *‘p’* value is smaller than 0.05.

## Results

All the 1200 patients, who presented to us were between 18 and 80 years of age, and 131 of these patients were taken into consideration as they presented within four days of fever and were confirmed to have dengue by the serological test dengue IgM antibody by the ELISA. The demographic details of the patients of this study is represented in Table 1. The highest number of dengue patients belonged to the working-age group and males, between the age group of 31 and 60 years (*n* = 131, 62.6%). Majority of the patients presented to us within the 3 days of fever (*n* = 131, 51.9%) at the time of admission. Among the 131 laboratory-confirmed cases, 44 (33.6%) were grouped as severe dengue cases as per the definition by the WHO, and the remaining 87 (66.4%), were non-severe dengue cases. The majority of severe dengue cases were aged between 31 and 60 years, and 63.4% of them were males.

**Table 1:** Baseline characteristics of dengue patients (*n* = 131)

Baseline characteristics	Number (*n* = 131)	Percentage	Severe dengue (*n* = 44)
*Age group (years)*			
< 30	42	32.1	13
31–60	82	62.6	26
> 60	7	5.3	5
*Sex*			
Male	83	63.4	25
Female	48	36.6	19
*Hospital stay*			
3–5	74	56.5	18
6–8	49	37.4	23
> 8	8	6.1	3
*Duration of fever (days)*			
Day 3	68	51.9	22
Day 4	63	48.1	22

The present study observed that, most of the patients were discharged from the hospital within the 5 days after the inpatient admission (*n* = 131, 56.5%). However, the mean duration of hospital stay of severe dengue cases was between 6 and 8 days.

Table 2 shows the correlation between the severity of dengue and various complications observed at the time of admission. The number of patients who developed more than one complication were 13 (29.5%). The serositis was found to be the most common complication among them (24.4%). There was a significant association (*p* < 0.005) between the severity of dengue and complications such as renal failure, shock, hepatitis, and serositis as per the chi-square test. Among the dengue victims who were under evaluation, edema of gall bladder existed in nearly 66 cases (Table 3). The 39 patients (89%), who had severe dengue (*n* = 44) revealed significant gall bladder wall thickening (Table 3), and this correlation was statistically significant (*p* < 0.000). The serum levels of AST, ferritin, platelet count, H score, total count, and hemoglobin were determined in severe dengue cases (Table 4) and they revealed elevated AST levels (mean = 407), followed by the elevated ferritin levels (mean = 9125 μg/l) and thrombocytopenia (mean = 26,182 μg/l). The mean Hb value was 13.12 gm%, and the mean total leukocyte count was found to be 4270. It is seen from Table 4 that, thrombocytopenia, rise in AST, H score, and ferritin levels have a highly significant positive correlation with the severity of dengue.

**Table 2:** Correlation between the severe form of dengue and its complications

Complications	Number (frequency)	*p* value
Serositis	32 (24.4%)**	< 0.01
Bleeding	04 (3.1%)**	< 0.01
ARDS	04 (3.1%)**	< 0.01
Renal failure	06 (4.6%)**	< 0.01
Encephalitis	01 (0.8%)*	> 0.01
Shock	08 (6.1%)**	< 0.01
Hepatitis	05 (3.8%)**	< 0.01
Myocarditis	01 (0.8%)*	> 0.01
More than 1 complication	13 (29.5%)**	< 0.01

**Statistically highly significant; *statistically not significant

**Table 3:** Correlation of gallbladder oedema with the blood investigation profile

Parameters	Gall bladder edema		Pearson chi square	*p* value
	Present	Absent		
Non-severe cases (*n* = 87, 66.4%)	27	60		
Severe cases (*n* = 44, 33.6%)	39	5	38.785	< 0.0001**
Total cases	66	65		
Mean AST	379.6	131.75		< 0.0001**
Mean platelet μg/l	23,378	63,230		< 0.0001**
Mean ferritin	9972	1768		< 0.0001**
Mean H score	169	113		< 0.0001**
Mean total count	4021	4264		> 0.01*

Mann–Whitney test; *p* value—**statistically highly significant; *statistically not significant

**Table 4:** Comparison of laboratory profile of severe and non-severe dengue cases

Variables	Severe dengue (*n* = 44)	Non-severe dengue (*n* = 87)	‘*p*’ value
	Mean ± standard deviation	Mean ± standard deviation	
AST	407.55 ± 67.9	180.87 ± 31.2	< 0.0001
Ferritin μg/l	9125.34 ± 124.53	4271.47 ± 491.3	< 0.01
Platelet	26,182 ± 248.65	51,736 ± 479.22	0.001
H score	155.41 ± 41	134.23 ± 43	< 0.01
TC	4270.45 ± 157.3	4077.47 ± 222.1	> 0.01

**Fig. 1:**
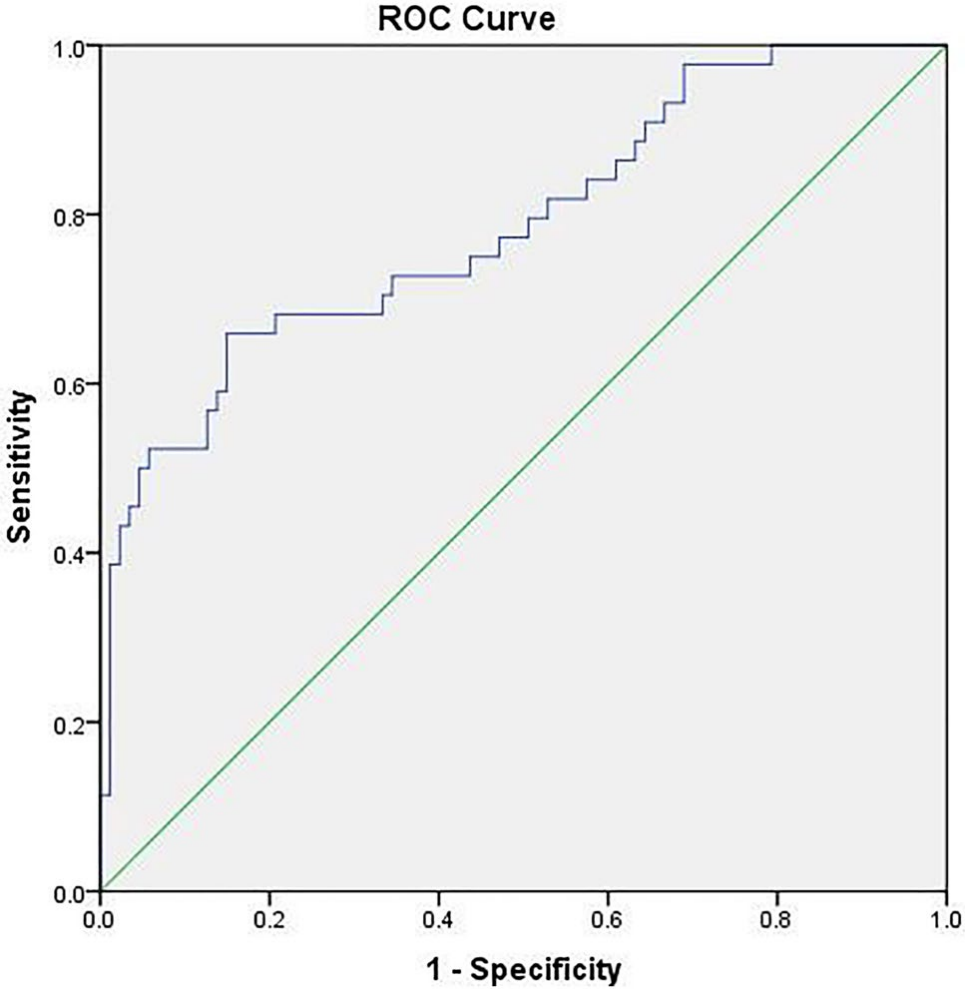
The sensitivity and specificity of serum ferritin in determining the severity of dengue (area under the curve; test result variable(s): ferritin; area − 0.788; std. error under the nonparametric assumption − 0.044; asymptotic sig. null hypothesis: true area = 0.5–0.003; Asymptotic 95% confidence interval lower bound − 0.702; upper bound − 0.873)

The receiver operating characteristic (ROC) curve analysis was performed to determine the ideal cut-off levels of ferritin in severe dengue cases (Fig. 1). The area in the ROC curve was 0.788, with a standard error of 0.44, as shown in the Fig. 1. The sensitivity and specificity of the severe dengue were 72.7 and 62.1, respectively. The asymptomatic 95% confidence interval was 0.702 (lower bound) and 0.873 (upper bound). The ‘*p* value’ was 0.003.

All the patients, who were included in this study were asked to come for the follow-up in the outpatient department after 2 and 4 weeks. However, the serial measurements of serum ferritin and ultrasonography were not performed because of the higher cost of these investigations. Most of our patients recovered well from our treatment and resumed back their duties, soon after the discharge from the hospital.

## Discussion

There has been a rise in the dengue incidence rate across the globe, in the recent years. One recent estimate indicates about 390 million dengue cases per year, of which 96 million cases manifest clinically (Bos et al 2018; Li and Wu 2015). Among them, about 5 lakh cases require hospitalization every year for the severe dengue, which has a mortality rate of about 2.5%. Dengue is basically a viral, infectious disease, which is transmitted by the female mosquito of the species, *A. aegypti* and less commonly by the Aedes albopictus. There has been a dramatic increase in the frequency of outbreaks in India in the last few decades. This disease is rampant throughout the world, especially in the tropics. Dengue causes a broad spectrum of disease severity ranging from the subclinical state to the severe form of the disease (Holmes and Twiddy 2003). In most of the states of India, dengue is almost an endemic disease. At present, all the four serotypes are seen in the circulation. However, the predominant serotype keeps changing. The WHO has classified the dengue into two major categories, non-severe variety with or without the warning signs and the severe variety. The non-severe type involves a high grade fever (104 °F or 40 °C), which is accompanied by any two of the following symptoms during the febrile phase—nausea, vomiting, rash, aches and pains, positive tourniquet test, and leukopenia. About 3–7 days after the onset of illness, fever drops to below 100 °F (38 °C) and the warning symptoms like tenderness in the abdomen, stomach burn, nausea, vomiting, mucosal bleed, lethargy, restlessness, hepatomegaly and rising hematocrit with thrombocytopenia start to appear, which require strict medical intervention. The inevitable cases progress towards severe dengue, which is evidenced by the respiratory distress, shock, massive bleeding, leakage of plasma, and organ impairment.

The earlier studies included few markers of severe form of dengue like plasmacytoid and dendritic cells (Pichyangkul et al. 2003), cytokines like IFN gamma (Tian et al. 2019), TNF alpha, IL 6 (Masood et al. 2018), IL 10 (Tian et al. 2019), MIF; chemokines like CXCL 10 (Masood et al. 2018); complements like C3a, C5a (King et al. 2020); proteases like tryptase and chymase (King et al. 2020). The endothelial activation markers include mediators of endothelial function like angiopoietin 1, angiopoietin 2 (Page and Liles 2013); components of coagulation system like von Willebrand factor (Page and Liles 2013); ADAMTS 13, thrombomodulin; cell surface adhesion molecules like sICAM, sVCAM; permeability mediators like VEGF (Page and Liles 2013), vascular endothelial growth factor receptor 1 (VEGFR1). The biochemical serum markers, which are routinely used in the laboratory include total cholesterol, HDL, LDL; lipopolysaccharides like lipopolysaccharide-binding protein (LPB), CD14; liver enzymes like AST, ALT; serine protease. The genetic markers are certain gene expressions like SMAD5, SLC4A4, PSPH, etc. and circulating cell-free DNA. However, these genetic markers are not widely used as they are expensive and require refined apparatus. The etiopathogenesis of dengue is not clearly understood as there are no excellent disease models (Cunha et al. 2022). There is a scarcity of animal experiments, which can produce dengue illness like the humans. Hence, clinical studies have been the key to understand the pathogenesis of dengue. Currently, there are no routine clinical or laboratory parameters to predict the severity of individual dengue cases, which require close monitoring (Srikiatkhachorn and Green 2010). It was reported that, the invention of diagnostics to identify the severe form of dengue is essential to decrease the morbidity and mortality rates (Wong et al. 2020).

In the present study, the difference in the patterns of serum ferritin level was studied in the patients with non-severe and severe dengue. The severe dengue patients had a mean ferritin level of 9125.34 μg/l, whereas, in the non-severe group, this was 4271 μg/l. This comparison was statistically significant as the ‘*p* value’ was 0.003. We could observe that the ROC curve analysis of serum ferritin level, as a marker in severe dengue exhibited an area of 0.788, with a standard error of 0.44. The ‘*p* value’ was 0.003, which suggests that serum ferritin level can be used as a marker of severe dengue. However, this finding is slightly different from that of Roy Chaudhuri et al. (2017), as our study had more non-severe dengue cases, whereas they had more number of severe dengue cases with the warning signs. It was reported that, patients below 593 ng/ml of ferritin levels are not at higher risk of developing low platelet count (Lodha et al. 2022). The earlier study from children, suggests that more elevated ferritin and IL-18 are associated with the severe dengue (Valero et al. 2019). Dengue causes an inflammatory state, which leads to non-remitting high fever, hepatosplenomegaly, hemorrhage, lymphadenopathy and CNS dysfunction. Since hemophagocytic syndrome (HS) may be difficult to distinguish from severe sepsis or flare of underlying autoimmune diseases, the availability of a simple score known as ‘the H score’, which allows the clinician to predict it in the patients and plan the appropriate treatment decisions at the earliest. The H score is a very well validated score devoted to the diagnosis of reactive HS, which consists of clinical, biologic and cytologic variables, which are appropriately weighted. The variables included in calculating the H score include known underlying immunosuppression, temperature, organomegaly, cytopenias, ferritin, triglyceride, fibrinogen, serum glutamic oxaloacetic transaminase and hemophagocytosis features on bone marrow aspirate. In our study, the calculated mean H score among the severe dengue patients was 155 ± 41, which was statistically significant, whereas, in non-severe dengue, the score was 134 ± 43. The ‘H’ score was proven to have a good performance in both developmental and validation data sets, which ensures that it is correctly calibrated to the range of diseases associated with the HS. This present study observed that, dengue has a high frequency of prevalence in the age group between 30 and 60 years than in the age below 30 years. Initially, dengue was considered to be a disease of children, but now it is very prevalent in adults and elderly individuals (Mallhi et al. 2015; Guerra-Silveira and Abad-Franch 2013). Increase in the movement of adult population, greater access to the health care facilities, and reporting of causal factors of dengue to the healthcare workers might be the reason for this high incidence of dengue infection among the adults. Among the adults aged 30–60 years, the higher severity of dengue was found, with about 59% of the severe cases belonging to this age group (Table 1). It would most probably have attributed to the presence of secondary infections and the likelihood of previous exposure to the dengue infection, leading to the increased severity among this population (Cardier et al. 2005). This current study observed that, males are more commonly affected by the dengue virus than the females. However, there was no statistically significant difference observed in this comparison. This was also observed in the study by Anker and Arima (2011), but in contrast to a study done by Murugananthan et al. (2014). The fever of dengue patients usually last about 2–7 days (Mallhi et al. 2015). In this study, patients who presented with less than 4 days of febrile illness only were included to look for the early predictors of the severity of the disease. Most of the patients (57%) had a hospital stay of 3–5 days, which is similar to the study by Mallhi et al. (2015). The prolonged hospital stay for more than 5 days was consistent with the severity of disease, amounting to 59% of the severe dengue cases. This also imposes a significant liability on the current health care.

The involvement of liver, brain, kidney, and heart were previously reported in severe dengue cases (Chaiyaratana et al. 2008). The liver involvement revealed elevated levels of liver function tests. Several studies have reported elevation in both the AST and ALT, as the predictors of severe dengue. Mean levels of AST were significantly higher among the severe dengue cases in our study (Table 4) and are more predictive of severe bleeding outcomes, according to another study. In addition, hepatomegaly accounted for 75% of the severe dengue cases (Table 2). The dengue virus directly affects the Kupffer cells of the liver resulting in the elevation of transaminases and liver enlargement. The liver damage in dengue infection is attributed to the activation of the cell-mediated immune system and is evident more in the severe dengue cases. The history of spontaneous bleeding was present in 9% of our severe dengue cases, which suggests the extent of liver cell injury. The spectrum of dengue-induced renal injury can range from mild proteinuria to severe acute kidney injury. The renal failure was found to be present in 13.6% of the cases with severe dengue (Table 2). The multi-organ dysfunction syndrome can cause various organ bleeds, leading to the increased mortality in severe dengue. In this study, the various complications present were serositis (24%), bleeding manifestations (3.1%), acute respiratory distress syndrome (3.1%), renal failure (4.6%), encephalitis (0.8%), shock (6.1%), hepatitis (3.8%) and myocarditis (0.8%). The minor bleeding was due to mucosal bleed and is a useful warning sign for the progression of disease to the severity and WHO and TDR proposed this in 2009. Major bleeding could be due to the low platelet count or prolongation of the aPTT. This study’s hematological profile of dengue patients was taken into consideration and compared to differentiate the severe dengue from the non-severe dengue cases. Thrombocytopenia was more common among the severe dengue patients, and this finding is consistent with the previous reports (Boo et al. 2019). This is predominantly due to the increased destruction of platelets as a result of the compliment activation, peripheral sequestration, and marrow dysfunction, and, of late, oxidative stress (Soundravally et al. 2015). The platelets are also shown to adhere to the endothelial cells due to stimulation by the dengue virus (Krishnamurti et al. 2002).

In this present study, hyperferritinemia was associated with the low platelet count. Thrombocytopenia is a classical of dengue infection, and this can be caused due to binding of platelets to the activated endothelial cells (Teixeira et al. 2008). It was reported that the edema of the gall bladder is associated with the dengue hemorrhagic fever, so every dengue patient should be screened for it (Adil et al. 2020). The sensitivity and specificity of the thickness of the gall bladder in assessing the admission to the intensive care unit or general ward were 100% and 62.1% (Ibrahim et al. 2022). The thickening of gallbladder, which is detected radiologically, can predict the disease progression in ambulatory patients in the early stages of dengue (Gleeson et al. 2022). The gall bladder wall thickness was promising as a predictive test for the severe dengue on day 3 or 4, the time before the onset of the critical phase in the pediatric population. Colbert et al. (2007) showed a high negative predictive value for detecting the DHF/DSS, which demonstrated the use of ultrasound reliably in the outpatient and inpatient settings in triage to rule out the severe dengue. This also helps to determine, which patients need to be monitored closely and whom to hospitalize. The gall bladder wall edema on the 3rd to 4th day was associated with the severe dengue in the present study. However, in Michels et al. (2013) study, the gall bladder wall edema was associated with severe dengue on the 7th day of illness. The honeycomb morphology of the gall bladder was observed more in the extreme form of dengue. The deteriorating cases of dengue showed a trend in changing from uniform thickening of the gall bladder wall to a honeycomb morphology during the later stages of fever. There was an increase in the gall bladder wall thickness from the 2nd day to the 5th day. The cases without the initial warning signs on the 2nd day of fever progressed to the warning signs on the 3rd day and eventually moved to the severe form of dengue on the 5th day.

The significance of gall bladder edema in dengue cases was proven in previous studies, and it was shown that it has a prognostic value. Based on the ultrasound examination, the current study also looked at gall bladder wall edema and its differences in the non-severe and severe dengue patients. This showed that significant gall bladder thickening was present in 89% of the severe dengue cases, whereas this was seen in only 31% of the non-severe dengue cases. The presence of gall bladder wall edema was also proved to have a prognostic significance in various conditions like hypoalbuminemia, cholecystitis (Handler 1979), acute hepatitis (Jüttner et al. 1982) and liver cirrhosis (Wang et al. 1997). Therefore, diffuse gall bladder wall thickening is also seen in various other conditions, and its exact cause is usually determined by the correlation of the associated clinical findings and imaging features.

This study has certain limitations, like the inclusion of patients with a history of less than 4 days of febrile illness only. This may add bias into the research, and could be one of the reason for the higher numbers of severe dengue cases seen in this study. There was also an association between the severity of dengue and complications such as renal failure, shock, hepatitis, and serositis, when evaluated. The present study has the rationale of selecting the ferritin and edema of the gall bladder as the early diagnostic markers in predicting the severe form of dengue. But neither ferritin nor the gall bladder edema is specific to the dengue infection. The ferritin levels are also elevated in other infectious diseases like SARS-CoV-2 (Mahroum et al. 2022), and gall bladder edema is not specific to the dengue. These can be considered as the drawbacks of this present study.

## Conclusion

In this present study, we found that thrombocytopenia, rising ferritin, AST, and H score positively correlated with the presence of gall bladder wall edema in severe dengue patients. However, increasing serum ferritin levels and diffuse gall bladder wall thickening are also seen in other various conditions. Their exact cause has to be determined by correlating the associated clinical findings and imaging features.

## References

[CR1] Adil B, Rabbani A, Ahmed S, Arshad I, Khalid MA (2020). Gall bladder wall thickening in dengue fever—aid in labelling dengue hemorrhagic fever and a marker of severity. Cureus.

[CR2] Anker M, Arima Y (2011). Male-female differences in the number of reported incident dengue fever cases in six Asian countries. Western Pac Surveill Response J.

[CR3] Avirutnan P, Punyadee N, Noisakran S, Komoltri C, Thiemmeca S, Auethavornanan K, Jairungsri A, Kanlaya R, Tangthawornchaikul N, Puttikhunt C, Pattanakitsakul SN, Yenchitsomanus PT, Mongkolsapaya J, Kasinrerk W, Sittisombut N, Husmann M, Blettner M, Vasanawathana S, Bhakdi S, Malasit P (2006). Vascular leakage in severe dengue virus infections: a potential role for the nonstructural viral protein NS1 and complement. J Infect Dis.

[CR4] Avirutnan P, Zhang L, Punyadee N, Manuyakorn A, Puttikhunt C, Kasinrerk W, Malasit P, Atkinson JP, Diamond MS (2007). Secreted NS1 of dengue virus attaches to the surface of cells via interactions with heparan sulfate and chondroitin sulfate E. PLoS Pathog.

[CR5] Avirutnan P, Fuchs A, Hauhart RE, Somnuke P, Youn S, Diamond MS, Atkinson JP (2010). Antagonism of the complement component C4 by flavivirus nonstructural protein NS1. J Exp Med.

[CR6] Boo YL, Lim SY, P'ng HS, Liam C, Huan NC, (2019). Persistent thrombocytopenia following dengue fever: what should we do?. Malays Fam Physician.

[CR7] Bos S, Gadea G, Despres P (2018). Dengue: a growing threat requiring vaccine development for disease prevention. Pathog Glob Health.

[CR8] Bosch I, Xhaja K, Estevez L, Raines G, Melichar H, Warke RV, Fournier MV, Ennis FA, Rothman AL (2002). Increased production of interleukin-8 in primary human monocytes and in human epithelial and endothelial cell lines after dengue virus challenge. J Virol.

[CR9] Cardier JE, Mariño E, Romano E, Taylor P, Liprandi F, Bosch N, Rothman AL (2005). Proinflammatory factors present in sera from patients with acute dengue infection induce activation and apoptosis of human microvascular endothelial cells: possible role of TNF-alpha in endothelial cell damage in dengue. Cytokine.

[CR10] Carr JM, Hocking H, Bunting K, Wright PJ, Davidson A, Gamble J, Burrell CJ, Li P (2003). Supernatants from dengue virus type-2 infected macrophages induce permeability changes in endothelial cell monolayers. J Med Virol.

[CR11] Chaiyaratana W, Chuansumrit A, Atamasirikul K, Tangnararatchakit K (2008). Serum ferritin levels in children with dengue infection. Southeast Asian J Trop Med Public Health.

[CR12] Chotiwan N, Brito-Sierra CA, Ramirez G, Lian E, Grabowski JM, Graham B, Hill CA, Perera R (2022). Expression of fatty acid synthase genes and their role in development and arboviral infection of *Aedes aegypti*. Parasit Vectors.

[CR13] Colbert JA, Gordon A, Roxelin R, Silva S, Silva J, Rocha C, Harris E (2007). Ultrasound measurement of gallbladder wall thickening as a diagnostic test and prognostic indicator for severe dengue in pediatric patients. Pediatr Infect Dis J.

[CR14] Cunha MS, de Moura CT, Guerra JM, Ponce CC, Fernandes NCCA, Résio RA, Claro IM, Salles F, Lima Neto DF, Sabino E (2022). A fatal case of dengue hemorrhagic fever associated with dengue virus 4 (DENV-4) in Brazil: genomic and histopathological findings. Braz J Microbiol.

[CR15] Deubel V, Laille M, Hugnot JP, Chungue E, Guesdon JL, Drouet MT, Bassot S, Chevrier D (1990). Identification of dengue sequences by genomic amplification: rapid diagnosis of dengue virus serotypes in peripheral blood. J Virol Methods.

[CR16] Fonseca-Portilla R, Martínez-Gil M, Morgenstern-Kaplan D (2021). Risk factors for hospitalization and mortality due to dengue fever in a Mexican population: a retrospective cohort study. Int J Infect Dis.

[CR17] García-Carreras B, Yang B, Grabowski MK, Sheppard LW, Huang AT, Salje H, Clapham HE, Iamsirithaworn S, Doung-Ngern P, Lessler J, Cummings DAT (2022). Periodic synchronisation of dengue epidemics in Thailand over the last 5 decades driven by temperature and immunity. PLoS Biol.

[CR18] Gleeson T, Pagnarith Y, Habsreng E, Lindsay R, Hill M, Sanseverino A, Patel V, Gaspari R (2022). Dengue management in triage using ultrasound in children from Cambodia: a prospective cohort study. Lancet Reg Health West Pac.

[CR19] Guerra-Silveira F, Abad-Franch F (2013). Sex bias in infectious disease epidemiology: patterns and processes. PLoS ONE.

[CR20] Handler SJ (1979). Ultrasound of gallbladder wall thickening and its relation to cholecystitis. Am J Roentgenol.

[CR21] Holmes EC, Twiddy SS (2003). The origin, emergence and evolutionary genetics of dengue virus. Infect Genet Evol.

[CR22] Ibrahim MA, Hamzah SS, Md Noor J, Mohamad MIK, Mokhtar MF, Isa MR, Abdul Rani MF (2022). The association of ultrasound assessment of gallbladder wall thickness with dengue fever severity. Ultrasound J.

[CR23] Jüttner H, Ralls P, Quinn M, Jenney J (1982). Thickening of the gallbladder wall in acute hepatitis: ultrasound demonstration. Radiology.

[CR24] King CA, Wegman AD, Endy TP (2020). Mobilization and activation of the innate immune response to dengue virus. Front Cell Infect Microbiol.

[CR25] Krishnamurti C, Peat RA, Cutting MA, Rothwell SW (2002). Platelet adhesion to dengue-2 virus-infected endothelial cells. Am J Trop Med Hyg.

[CR26] Li Y, Wu S (2015). Dengue: what it is and why there is more. Sci Bull Sci Found Philipp.

[CR27] Libraty DH, Endy TP, Houng HS, Green S, Kalayanarooj S, Suntayakorn S, Chansiriwongs W, Vaughn DW, Nisalak A, Ennis FA, Rothman AL (2002). Differing influences of virus burden and immune activation on disease severity in secondary dengue-3 virus infections. J Infect Dis.

[CR28] Lodha A, Pillai A, Reddy P, Munshi N (2022). Using first-contact serum ferritin to predict severe thrombocytopenia in dengue patients: determination and validation in independent cohorts. Infect Dis (lond).

[CR29] Mahroum N, Alghory A, Kiyak Z, Alwani A, Seida R, Alrais M, Shoenfeld Y (2022). Ferritin - from iron, through inflammation and autoimmunity, to COVID-19. J Autoimmun.

[CR30] Mallhi TH, Khan AH, Adnan AS, Sarriff A, Khan YH, Jummaat F (2015). Clinico-laboratory spectrum of dengue viral infection and risk factors associated with dengue hemorrhagic fever: a retrospective study. BMC Infect Dis.

[CR31] Masood KI, Jamil B, Rahim M, Islam M, Farhan M, Hasan Z (2018). Role of TNF α, IL-6 and CXCL10 in dengue disease severity. Iran J Microbiol.

[CR32] Medin CL, Fitzgerald KA, Rothman AL (2005). Dengue virus nonstructural protein NS5 induces interleukin-8 transcription and secretion. J Virol.

[CR33] Michels M, Sumardi U, de Mast Q, Jusuf H, Puspita M, Dewi IM, Sinarta S, Alisjahbana B, van der Ven AJ (2013). The predictive diagnostic value of serial daily bedside ultrasonography for severe dengue in Indonesian adults. PLoS Negl Trop Dis.

[CR34] Murugananthan K, Kandasamy M, Rajeshkannan N, Noordeen F (2014). Demographic and clinical features of suspected dengue and dengue haemorrhagic fever in the Northern Province of Sri Lanka, a region afflicted by an internal conflict for more than 30 years—a retrospective analysis. Int J Infect Dis.

[CR35] Page AV, Liles WC (2013). Biomarkers of endothelial activation/dysfunction in infectious diseases. Virulence.

[CR36] Parmar JP, Mohan C, Vora M (2017). Patterns of gall bladder wall thickening in dengue fever: a mirror of the severity of disease. Ultrasound Int Open.

[CR37] Petchiappan V, Hussain TM, Thangavelu S (2019). Can serum ferritin levels predict the severity of dengue early: an observational study. Int J Res Med Sci.

[CR38] Pichyangkul S, Endy TP, Kalayanarooj S, Nisalak A, Yongvanitchit K, Green S, Rothman AL, Ennis FA, Libraty DH (2003). A blunted blood plasmacytoid dendritic cell response to an acute systemic viral infection is associated with increased disease severity. J Immunol.

[CR39] Roy Chaudhuri S, Bhattacharya S, Chakraborty M, Bhattacharjee K (2017). Serum ferritin: a backstage weapon in diagnosis of dengue fever. Interdiscip Perspect Infect Dis.

[CR40] Ruddell RG, Hoang-Le D, Barwood JM, Rutherford PS, Piva TJ, Watters DJ, Santambrogio P, Arosio P, Ramm GA (2009). Ferritin functions as a proinflammatory cytokine via iron-independent protein kinase C zeta/nuclear factor kappaB-regulated signaling in rat hepatic stellate cells. Hepatology.

[CR41] Senjo H, Higuchi T, Okada S, Takahashi O (2018). Hyperferritinemia: causes and significance in a general hospital. Hematology.

[CR42] Songjaeng A, Thiemmeca S, Mairiang D, Punyadee N, Kongmanas K, Hansuealueang P, Tangthawornchaikul N, Duangchinda T, Mongkolsapaya J, Sriruksa K, Limpitikul W, Malasit P, Avirutnan P (2022). Development of a singleplex real-time reverse transcriptase PCR assay for pan-dengue virus detection and quantification. Viruses.

[CR43] Soundravally R, Agieshkumar B, Daisy M, Sherin J, Cleetus C (2015). Ferritin levels predict severe dengue. Infection.

[CR44] Srikiatkhachorn A, Green S, Rothman AL (2010). Markers of dengue disease severity. Dengue virus.

[CR45] Teixeira MG, Costa MC, Coelho G, Barreto ML (2008). Recent shift in age pattern of dengue hemorrhagic fever. Brazil Emerg Infect Dis.

[CR46] Tian Y, Seumois G, De-Oliveira-Pinto LM, Mateus J, Herrera-de la Mata S, Kim C, Hinz D, Goonawardhana NDS, de Silva AD, Premawansa S, Premawansa G, Wijewickrama A, Balmaseda A, Grifoni A, Vijayanand P, Harris E, Peters B, Sette A, Weiskopf D (2019). Molecular signatures of dengue virus-specific IL-10/IFN-γ co-producing CD4 T cells and their association with dengue disease. Cell Rep.

[CR47] Valero N, Mosquera J, Torres M, Duran A, Velastegui M, Reyes J, Fernandez M, Fernandez G, Veliz T (2019). Increased serum ferritin and interleukin-18 levels in children with dengue. Braz J Microbiol.

[CR48] van de Weg CA, Huits RM, Pannuti CS, Brouns RM, van den Berg RW, van den Ham HJ, Martina BE, Osterhaus AD, Netea MG, Meijers JC, van Gorp EC, Kallas EG (2014). Hyperferritinaemia in dengue virus infected patients is associated with immune activation and coagulation disturbances. PLoS Negl Trop Dis.

[CR49] Vaughn DW, Green S, Kalayanarooj S, Innis BL, Nimmannitya S, Suntayakorn S, Endy TP, Raengsakulrach B, Rothman AL, Ennis FA, Nisalak A (2000). Dengue viremia titer, antibody response pattern, and virus serotype correlate with disease severity. J Infect Dis.

[CR50] Wang TF, Hwang SJ, Lee EY, Tsai YT, Lin HC, Li CP, Cheng HM, Liu HJ, Wang SS, Lee SD (1997). Gall-bladder wall thickening in patients with liver cirrhosis. J Gastroenterol Hepatol.

[CR51] Wang W, Knovich MA, Coffman LG, Torti FM, Torti SV (2010). Serum ferritin: past, present and future. Biochim Biophys Acta.

[CR52] Wong PF, Wong LP, AbuBakar S (2020). Diagnosis of severe dengue: challenges, needs and opportunities. J Infect Public Health.

[CR53] Zhang XB, Fei YX, He T, Gao L, Zhang YT, Gao YD, Li G, Wang J, Ru QJ, Wang HQ, Chen GY (2021). Correlation analysis between serum ferritin level and liver damage in acute stage of dengue fever. Zhonghua Gan Zang Bing Za Zhi.

